# The human cerebellum has almost 80% of the surface area of the neocortex

**DOI:** 10.1073/pnas.2002896117

**Published:** 2020-07-28

**Authors:** Martin I. Sereno, Jörn Diedrichsen, Mohamed Tachrount, Guilherme Testa-Silva, Helen d’Arceuil, Chris De Zeeuw

**Affiliations:** ^a^Experimental Psychology, University College London, London WC1H 0AP, United Kingdom;; ^b^Department of Psychological Sciences, Birkbeck University of London, London WC1E 7HX, United Kingdom;; ^c^Department of Psychology, San Diego State University, San Diego, CA 92182;; ^d^Institute of Cognitive Neuroscience, University College London, London WC1N 3AR, United Kingdom;; ^e^Departments for Computer Science and Statistics, University of Western Ontario, London N6A 3K7, ON, Canada;; ^f^UCL Institute of Neurology, University College London, London WC1N 1PJ, United Kingdom;; ^g^Nuffield Department of Clinical Neurosciences, University of Oxford, Oxford OX3 9DU, United Kingdom;; ^h^Department of Chemisty and Chemical Biology, Harvard University, Cambridge, MA 02138;; ^i^Athinoula A. Martinos Center for Biomedical Imaging, Massachusetts General Hospital, Charlestown, MA 02129;; ^j^Department of Neuroscience, Netherlands Institute for Neuroscience, Amsterdam NL-3000 CA, The Netherlands

**Keywords:** cerebellum, surface area, computational, unfolding, evolution

## Abstract

The surface of the human cerebellar cortex is much more tightly folded than the cerebral cortex. It was computationally reconstructed for the first time to the level of all individual folia from multicontrast high-resolution postmortem MRI scans. Its total shrinkage-corrected surface area (1,590 cm^2^) was larger than expected or previously reported, equal to 78% of the total surface area of the human neocortex. The unfolded and flattened surface comprised a narrow strip 10 cm wide but almost 1 m long. By applying the same methods to the neocortex and cerebellum of the macaque monkey, we found that its cerebellum was relatively much smaller, approximately 33% of the total surface area of its neocortex. This suggests a prominent role for the cerebellum in the evolution of distinctively human behaviors and cognition.

## Significance

The cerebellum has long been recognized as a partner of the cerebral cortex, and both have expanded greatly in human evolution. The thin cerebellar cortex is even more tightly folded than the cerebral cortex. By scanning a human cerebellum specimen at ultra-high magnetic fields, we were able to computationally reconstruct its surface down to the level of the smallest folds, revealing that the cerebellar cortex has almost 80% of the surface area of the cerebral cortex. By performing the same procedure on a monkey brain, we found that the surface area of the human cerebellum has expanded even more than that of the human cerebral cortex, suggesting a role in characteristically human behaviors, such as toolmaking and language.

During human evolution, the cerebellum has expanded in parallel with the neocortex, especially in regions connected to frontal and parietal association areas (1, 2). Indeed, the changes in the cerebellum in terms of volume and neuron number have outstripped increases in the neocortex (3, 4). These evolutionary insights along with neuropsychological investigations have led to a reevaluation of the role of the cerebellum in human cognition (5–7).

Like the neocortex, the human cerebellum is a thin sheet of neuronal tissue intricately folded to reduce its overall volume while maintaining its two-dimensional (2D) topology. However, unlike the human neocortex, the surface of the human cerebellum has never been computationally reconstructed to the level of all individual small folds (folia). The cerebellar cortex is even thinner than the cerebral cortex and has predominantly mediolaterally oriented folds that pack its large anterior-posterior extent into a very compact volume. This anisotropy is due to the structural scaffold of parallel fibers that run in a mediolateral direction, perpendicularly intersecting the anteroposteriorly (sagittally) oriented sheet-like dendritic trees of Purkinje cells. Approximately 10 to 15 Purkinje cells arranged in a sagittal microzone receive the same climbing fiber input and form a basic unit of cerebellar computation (8). The mediolateral extent of the folia has expanded somewhat in evolution; however, perhaps due to limits on the length of parallel fibers, the expansion in the anteroposterior direction has been much greater—in humans, almost two orders of magnitude greater. This picture contrasts with the more nearly isotropic expansion of the human neocortex.

Our interest in creating an accurate folia-level surface reconstruction was particularly driven by the results of finely detailed (60+ sites/mm^2^) microelectrode mapping of the granule cell layer in somatosensory input regions in the cerebellum (9, 10), which revealed a unique “fractured somatotopy” in which tiny, internally somatotopic 2D patches of the body surface were arranged into a mosaic that routinely juxtaposes distant body parts to a much greater degree than is the case in primary somatosensory cortex. These intricately “fractured” maps were also detected in the Purkinje cell layer using localized naturalistic stimulation of the skin (11), likely the result of the higher synaptic density per Purkinje cell of the ascending limb of a parallel fiber compared with the more obvious mediolateral parallel fiber itself. During active movements involving multiple body parts and skin contact points, parallel fibers that extend across multiple patches can integrate information from many distant body parts to speed, refine, and coordinate complex actions. Future studies of the spatial arrangement of these fractured functional maps on the cerebellar surface, and their implications for the computational role of the cerebellum, including possible analogs in the “cognitive cerebellum,” will first require a detailed quantitative representation of the complexly folded surface of the human cerebellum.

## Results

We set out to reconstruct, unfold, and flatten the entire human cerebellar surface down to the level of individual folia to quantitatively measure its total surface area, better characterize its regional structure, and provide a full-resolution human cerebellar base map. Although neuroanatomists have long made diagrammatic reconstructions of the cerebellum, the landmark paper by Sultan and Braitenberg (12) provided the first complete manual reconstructions of the cerebellar surfaces of humans and many other species using stereologic methods based on ruler measurements of selected folia widths and lengths made on sample tissue blocks. The first detailed surface-based computational MRI reconstruction of a living human cerebellum used repeated scans of a single subject that were weighted for longitudinal relaxation time (T1), using 1-mm-wide voxels at 1.5 T (13). However, since a folium is typically only a few mm across, and because folia are so tightly packed against one another, partial volume effects made it difficult to fully resolve them. More recently, 7-T scans with slightly smaller voxels were used to reconstruct the lobular surfaces of individual cerebella (14), but partial volume effects again prevented complete recovery of all folia. As a pragmatic solution for the display of in vivo functional and structural data on the cerebellum, it has become common practice to use a surface that captures the average convex hull of the cerebellum and abstracts over the finer lobule- and folia-level details (15, 16).

**Fig. 1.**
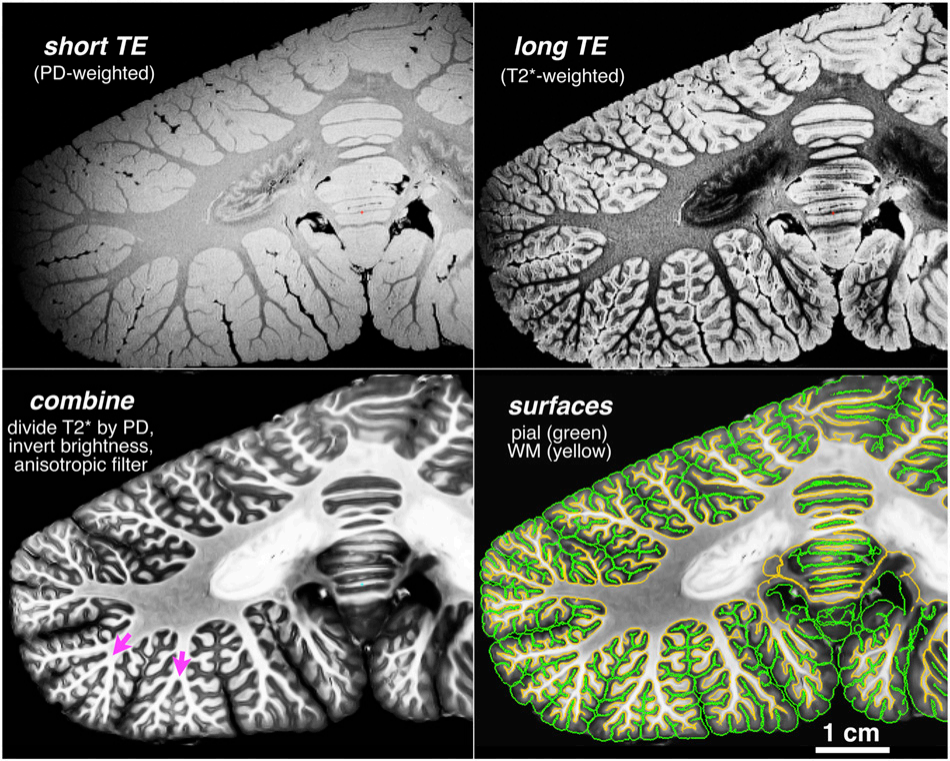
Coronal high-resolution MRI images of the human cerebellum. Short- and long-TE 9.4-T scans with 0.19-mm-wide isotropic voxels were combined and filtered to reconstruct the cerebellar gray/white (yellow) and pial (green) surfaces (*Lower Right*).

To provide a complete, quantitative, high-resolution reconstruction of the entire human cerebellar cortical surface recovering all folia, we scanned preserved cerebellar specimens at 9.4 T with much smaller isotropic 0.19-mm-wide voxels at two echo times (TEs) (Fig. 1): a short TE to generate proton density-weighted (PD) images and a long TE for images weighted by transverse relaxation time (T2*). We combined the images to cancel inhomogeneous coil receive fields, further normalized the result using analysis of functional neuroimages (AFNI) utilities, contrast-inverted it, and then extended the original FreeSurfer software to reconstruct, unfold, and flatten the entire cerebellar cortex (*SI Appendix*, *Supplementary Text*). Our estimate of the total cerebellar surface area was larger than any previous estimate, at 78% of the surface area of the entire human neocortex.

The cerebellum has two distinct hierarchical levels of folding: large lobules and small folia. These two levels of folding can be conveniently computationally distinguished in FreeSurfer. During surface reconstruction, FreeSurfer calculates two vertexwise quantities: (i) the local surface convexity (or concavity) at each vertex, calculated from the relative positions of neighboring vertices (“curv”), which marks the crown of each folium green and the intervening concave regions red, and (ii) the average convexity, the summed perpendicular distance that each vertex has moved during the local geometry-preserving unfolding/inflation process (“sulc”), which marks the crowns of the main lobules green (they move less) and the deep concave fissures (sulci) between them red (they move more) while mostly ignoring the folia-level details. Some ambiguity remains in cases where a main lobule divides into two or three sublobules (Fig. 1, *Bottom Left*, magenta arrows), in which case each sublobule will be separated by thinner red bands.

To determine the maximum voxel size capable of recovering the features of the human cerebellar cortex down to the level of individual folia, the final processed three-dimensional (3D) dataset was repeatedly down-sampled and was re-reconstructed, and then the resulting surface areas were compared with the surface area of the native 0.19-mm-wide voxel reconstruction. Downsampling to 0.21-mm voxels resulted in only a 1% loss of surface area, but 0.28-mm voxels caused a 14% loss, and 0.50-mm voxels caused a 50% loss (*SI Appendix*, *Supplementary Text*). This suggests that a full recovery of the human cerebellar surface to the level of individual folia requires voxels almost as small as those used here (∼150 voxels per mm^3^), which resulted in a dense surface tessellation with almost 5 million vertices, 25 times more than in a typical FreeSurfer cerebral hemisphere. The proton density images were used by themselves to separately reconstruct the left and right dentate nuclei (because the T2*/PD operation reduced the contrast in the dentate nuclei).

**Fig. 2.**
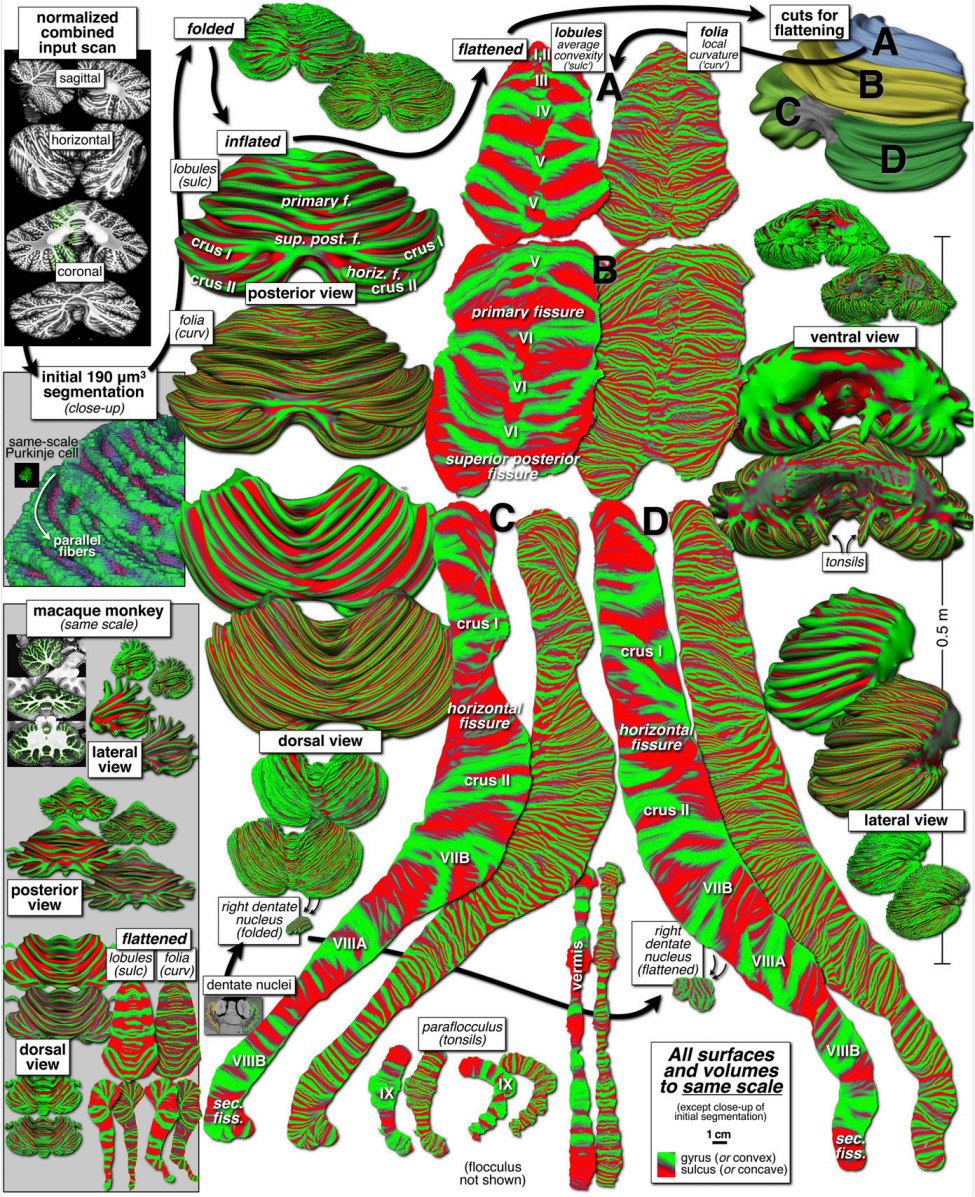
Cerebellar slices and folded, inflated, flattened cerebellar surfaces, all at same scale. Starting in the *Upper Left*, slice images are tessellated and the resulting surface was unfolded, cut (*Upper Right*), and flattened. Each surface is shown twice, first color-coded by FreeSurfer average convexity (“sulc,” which marks lobules) and then by local curvature (“curv,” which marks much smaller folia). At *Lower Left*, a macaque monkey cerebellum is shown at the same scale.

For ease of comparison, the input slice data and the folded, unfolded, and flattened surfaces (including the right dentate nucleus) are all illustrated at the same scale in Fig. 2. Each folded, unfolded, and flattened view is illustrated twice, once displaying the vertexwise value of the average convexity indicating the different lobules and then again (immediately to the right/below) displaying the local curvature, which visualizes the folding at the level of folia. Green surface patches indicate the crowns of lobules and the crowns of the folia, respectively. Movie S1 dynamically illustrates how the lobules come into view as unfolding proceeds.

**Fig. 3.**
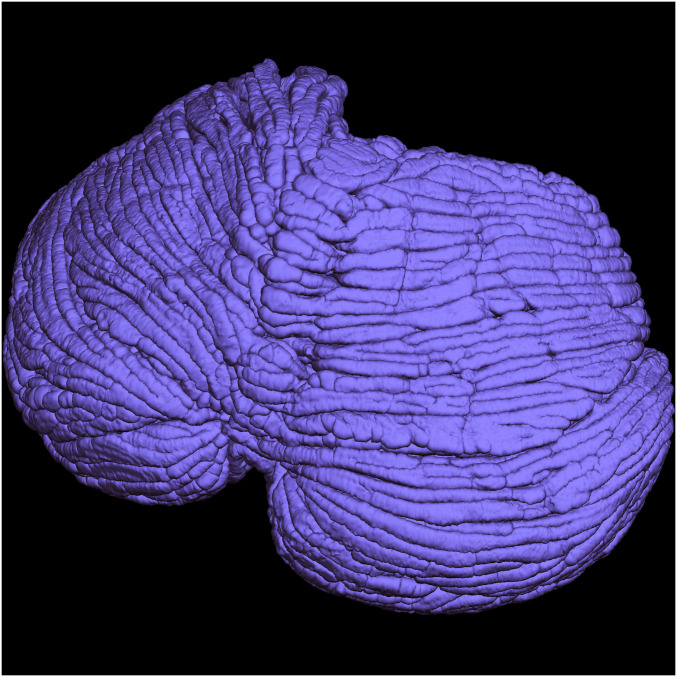
Reconstruction of the pial surface of the human cerebellum in superoposterior view.

The total area of the reconstructed pial surface of the cerebellar cortex (Fig. 3) was 1,590 cm^2^ after correcting for fixation-induced shrinkage (*SI Appendix*, *Supplementary Text*). This is considerably larger than any previous estimate. For comparison, the largest previous estimate was 1,128 cm^2^ reported by Sultan and Braitenberg (12). Previous MRI-based estimates from in vivo MRI scans were uniformly much smaller because individual folia were not completely resolved, as noted by the authors [e.g., Van Essen (13), 540 cm^2^; cerebellar high-resolution map (CHROMA) atlas external surface, 390 cm^2^; Diedrichsen and Zotow (16): gray/white matter surface, 125 cm^2^].

For comparison, the total area of the outer, pial surfaces of the left plus right cerebral hemispheres of female human subjects (which is 1.2 times the FreeSurfer “standard area” measured on the gray-white matter surface; *Materials and Methods*) is ∼2,038 cm^2^ (17). This means that the pial surface area of the human cerebellum is almost as large as that of the entire neocortex, even though the cerebellum is roughly one-eighth the volume of the neocortex.

Most anatomic studies of the cerebellum have used diagrammatic unfoldings and flattenings that do not uniformly minimize local surface distortion. It turned out to be surprisingly difficult to unfold and flatten the cerebellar cortical surface using local geometry-preserving methods developed for the neocortex, because the cerebellum has more Gaussian (intrinsic) curvature than the neocortex (13). At first, this might seem counterintuitive, because the elongated individual folia have a cylindrical shape (mostly extrinsic curvature) and thus should seemingly be able to be unfolded and flattened with little local areal/angular distortion (like unrolling a yoga mat). However, individual folia at the level of the midline of the cerebellum (vermis) clearly split up into multiple folia when they continue into the cerebellar hemispheres. At the lateral edges of the hemispheres, these folia appear in many cases to fuse again (Fig. 2, flat representation of folia). During the surface inflation process, this complex geometry leads to “bubbles” that are tied down at both the lateral edge and the midline (Fig. 2, ventral unfolded views and second half of Movie S1). Like a sphere, these lobular “bubbles” have a large amount of intrinsic curvature and cannot be further inflated (or flattened) without causing severe distortion.

Therefore, to flatten the surface without introducing severe local areal distortion, each mediolateral bubble was cut loose at its lateral ends in the anterior lobe and at both lateral and midline ends in the posterior lobe (e.g., crus I and II). Initial attempts to computationally unfurl the cut surface without further subdividing it were unsuccessful, because the local measurements that drive flattening were overwhelmed by the enormous size of the mesh. Therefore, the mesh was cut into four large pieces and three smaller pieces that were all flattened separately. The anterior part of the cerebellum was cut into two large pieces without a left/right cut (lobules I to V and lobules V and VI). At the large “superior posterior” fissure anterior to crus I, the surface was divided into left and right halves (including crus I, the horizontal fissure, crus II, VIIB, VIIIA, VIIIB, and the secondary fissure); the anteromedial end of each of these patches begins at two small paramedian regions where the white matter is exposed (no overlying folia). Finally, the two smaller paraflocculi (the “tonsils”; lobe IX), were flattened separately, as was the posterior vermis starting from the point where the posterior lobes were cut into right and left halves. Slight damage to the flocculi made full recovery of surface area there difficult.

After the cuts, the surface pieces could then be flattened while introducing only minimal local areal distortion. This resulted in an unfolded surface that was greatly extended in the anteroposterior direction; it was almost 1 m long, but only roughly 10 cm wide. The surface area of lobule VII together with lobule VIII was almost twice the total area of lobules I to VI. In contrast, in previous unfoldings (13), these two regions appear to have approximately equal areas because of the less complete recovery of folia in the complexly folded posterior cerebellum.

**Fig. 4.**
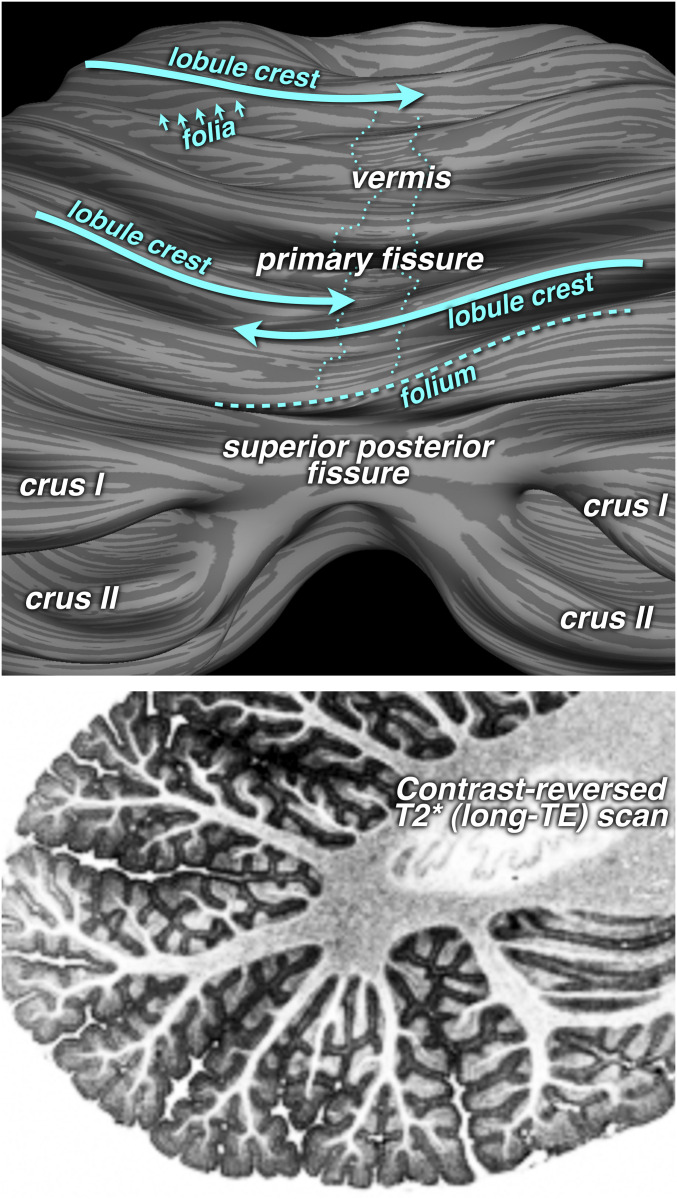
Detailed geometry of lobules and folia. Lobular crests sometimes turn into fissures across the midline (thick blue/cyan arrows). The axes of folia are often not parallel to lobular axes (small arrows). The dashed line (*Lower Middle*) outlines a single folium that spirals up from the depths of a fissure over the crest of lobule VI. The vermis is indicated by thin dotted lines. In the *Lower* scan (contrast-reversed long-TE, T2*-weighted), the granule cell (light gray) and molecular layers dark gray) can be distinguished from white matter (white).

Examination of the detailed geometry of the lobules and folia revealed several unexpected features. As (unfolded) lobules approached the midline, the crest of a lobule sometimes descended into a fissure in the opposite hemisphere (Fig. 4, large cyan arrows). In addition, although the long axes of the folia were sometimes approximately parallel to the long axis of a lobule, an inspection of the folia visualization (Fig. 2, folia/local curvature map; Fig. 4, small arrows) shows many regions in which the angle between the two is as large as 45° (e.g., lobule V). While rapidly paging through slices, it became apparent that individual folia often spiraled up from deep in a fissure to the crest of a lobule and then down the other side (Fig. 4, thick dashed line).

We also reconstructed the surfaces of the major output nuclei of the cerebellum, the dentate nuclei. Before unfolding, these nuclei have the approximate shape of ribbed pita pockets. The surfaces were flattened using a medial relaxation cut (Fig. 2, *Bottom*). Their total surface area (left and right) measured along their outer surfaces was 18.6 cm^2^; this does not include the much smaller interposed and fastigial nuclei. This gives an areal ratio of cerebellar cortex to cerebellar output nuclei of >80:1.

To provide a snapshot of how the surface area of the neocortex and cerebellum have changed in primate evolution, we reconstructed, measured, and unfolded the neocortical and cerebellar surfaces of a preserved macaque monkey brain using similar methods (Movie S2). A gadolinium-soaked macaque monkey brain was scanned with a standard FLASH sequence at 4.7 T using isotropic 0.15-mm^3^ voxels, as described previously (18). The reconstructed and inflated macaque monkey cerebellum (450 K vertices) is shown at the lower left of Fig. 2 at the same scale as the human cerebellum. The total shrinkage-corrected surface area of the pial surface of the macaque monkey cerebellum was 90 cm^2^. This is somewhat larger than has been reported previously [Sultan and Braitenberg (12), 81 cm^2^; Van Essen (13), 61 cm^2^]. For comparison, the total surface area of the pial surface of the macaque monkey neocortex from the same animal was 269 cm^2^. Thus, in macaque monkeys, the cerebellum has at least 33% of the surface area of the neocortex, while in humans, the cerebellum has ∼78% of the surface area of the neocortex, which explains why the complexity of folding of the human cerebellum has increased so dramatically. Although the voxel width was somewhat smaller for the monkey scan, the folia in the monkey are substantially smaller than the folia in humans, so our monkey cerebellar surface area estimate may be slightly too low. Nevertheless, we verified that all folia were recovered by inspecting the intersection of the surface with each slice.

## Discussion

By reconstructing the human cerebellar surface down to the level of individual folia, we found that its surface area was considerably larger than previously reported—amounting to 78% of the entire surface area of the human neocortex. Applying similar methods to the macaque monkey brain, we found that the surface area of its cerebellum is only approximately one-third of the surface area of its neocortex. This substantial relative increase in the surface area of the human cerebellum likely accommodates more extensive connections from parietal and prefrontal cortex and suggests that the large cerebellum may have been as important as a large neocortex for the origin of some distinctively human abilities (e.g., language, extensive toolmaking, complex sociality).

The high-resolution surface provides a quantitative folia-level base map for the human cerebellar cortex and is now publicly available. The complete surface can be used to estimate (noncontiguous) surface coverage of necessarily coarser functional voxels. It provides a veridical source space model for estimating scalp electric and magnetic potentials (19) and can serve as a ground truth for evaluating approximate cerebellar surface reconstructions from in vivo data. By combining quantitative mapping of gray matter myelination (20) with gray matter diffusion signature mapping (21), it may eventually be possible to visualize the sagittally oriented zonal structure of the human cerebellum in full detail. Finally, ultra-high field scans are within striking distance (22) of visualizing the fractured maps in the somatosensory cerebellum in vivo and, looking forward, possibly finding analogous structures in the “cognitive cerebellum” (expected size shown in *SI Appendix*, Fig. S1). The peculiar cerebellar style of computation, in which small internally organized patches from distant sources are brought together like a jumbled picture puzzle, might be able to help with higher-level computations, such as those involved in language or abstract reasoning. Rather than coordinating sensory signals to execute expert physical movements, parts of the cerebellum may have been extended in humans to help coordinate fictive “conceptual movements,” such as rapidly mentally rearranging a movement plan—or, in the fullness of time, perhaps even a mathematical equation.

## Materials and Methods

More detailed information is provided in *SI Appendix*, *Supplementary Text*.

### Specimen Preparation and Scan Parameters.

A female cerebellar specimen was scanned in a Fomblin-filled acrylic cylinder on a 9.4-T magnetic resonance scanner (Agilent Technologies) with short TE (PD-weighted) and long TE (T2*-weighted) 3D gradient echo (FLASH) sequences (PD: flip angle, 10°; TE, 3.7 ms; repetition time [TR], 15 ms; T2*: flip angle, 20°; TE, 18 ms; TR, 30 ms; matrix, 512 × 340 × 340; isotropic 0.19-mm-wide voxels; number of repetitions for each contrast, 10; total time, 12 h).

### Surface Reconstruction.

The cerebellar surface was reconstructed using csurf, an updated version of the original FreeSurfer surface reconstruction software that allows native use of 512^3^ datasets. The T2* image was normalized by dividing it by the PD image, further flattened with AFNI 3dUniformize, and brightness-inverted so white matter was lighter than gray matter. After applying a local (7 × 7 × 7 voxel) anisotropic filter in the plane of least brightness variation (Gaussian FWHM = 2.5 voxels = 0.40 mm width), the image was segmented, hand-edited, and tessellated to obtain an initial surface near the Purkinje cell layer containing 4.6M vertices (25 times more than in a standard FreeSurfer neocortical hemisphere). That initial surface was transformed into a gray/white matter surface and a non–self-intersecting pial surface by manipulating the image criterion for zero image error. Finally, the surface was cut to allow it to be flattened using FreeSurfer 5.3 mris_flatten.

### Measurement of Area.

Vertexwise areal measurements of the cerebellar pial surface were made after removing the surface covering the cut peduncles and were corrected for estimated fixation-induced shrinkage (3% volume isotropic). To estimate human female neocortical pial surface area, we scaled average in vivo female gray-white surface area taken from the literature using a gray/white-to-pial factor (1.2×) measured from 32 FreeSurfer 5.3 in-house reconstructed hemispheres.

### Deep Cerebellar Nuclei.

The deep cerebellar dentate nuclei were reconstructed and unfolded using similar methods directly from the PD images (the T2*/PD images had reduced contrast here).

### Macaque Monkey Surface Reconstructions.

The cerebellum and neocortical hemispheres of a macaque monkey were reconstructed and measured using similar methods from a T2*-weighted ex vivo 3D FLASH scan with isotropic 0.15 mm wide voxels, as described previously (18).

## Supplementary Material

Supplementary File

Supplementary File

Supplementary File

## Data Availability

Datasets including (i) original high-resolution isotropic 3D MRI data of the human cerebellum with two different contrasts; (ii) computationally combined, normalized, filtered, and edited versions of that 3D data; and (iii) FreeSurfer-compatible subject surfaces and vertexwise measurements reconstructed from the data can be downloaded from https://mri.sdsu.edu/sereno/cereb. Software for performing the analyses presented in this paper is available at https://mri.sdsu.edu/sereno/csurf or http://www.cogsci.ucsd.edu/∼sereno/.tmp/dist/csurf. We also used some of the utilities from the standard FreeSurfer 5.3 distribution (available at https://surfer.nmr.mgh.harvard.edu/) and from the AFNI distribution (available at https://afni.nimh.nih.gov/).
