# Supplementary material for: The human cerebellum has almost 80% of the surface area of the neocortex
Source: Proc Natl Acad Sci U S A. 2020 Jul 28;117(32):19538–43. doi: 10.1073/pnas.2002896117 (PMC7431020; doi:10.1073/pnas.2002896117)
Supplement: Supplementary File [file pnas.2002896117.sapp.pdf]

PNAS
www.pnas.org

Supplementary Information for:

The human cerebellum has almost 80% of the surface area of the neocortex

Martin I. Sereno, Jörn Diedrichsen, Mohamed Tachrount, Guilherme Testa-Silva, Helen d'Arceuil, Chris De Zeeuw

Corresponding author:
Martin I. Sereno
Email: msereno@sdsu.edu (or msereno@ucsd.edu)

This PDF file includes:

Legends for Movies S1 and S2
Supplementary Figures S1 and S2
Supplementary text
SI References

Other supplementary materials for this manuscript include the following:

Movies S1 and S2

www.pnas.org/cgi/doi/10.1073/pnas.2002896117

Supplementary Movies Legends (separate files)

Movie S1. Unfolding and refolding the reconstructed surface of the middle layers of human cerebellar cortex (movie loop). The initially folded reconstruction rotates, unfolds, and then refolds, first in posterior, then in anterior view. The deep fissures between the lobules are revealed as the surface unfolds. The near-midline cerebellar tonsils are visible in the anterior view (at 00:25 to 00:30). Because of the substantial intrinsic curvature of the cerebellar surface, the unfolded 'bubbles' formed by each lobule cannot be further unfolded/inflated without severe distortion.

Movie S2. Unfolding and refolding the reconstructed surface of the middle layers of macaque monkey cerebellar cortex (movie loop). The initially folded reconstruction rotates, unfolds, and refolds in parallel with the first movie (SI Appendix, Movie S1). By comparing the two movies, the huge expansion of the hemispheres relative to the vermis in humans, especially posteriorly, is quite apparent. Note that the much smaller monkey surface is *not* to same scale as the human surface (see Fig. 2 for true relative sizes of the surfaces).

Supplementary Figures

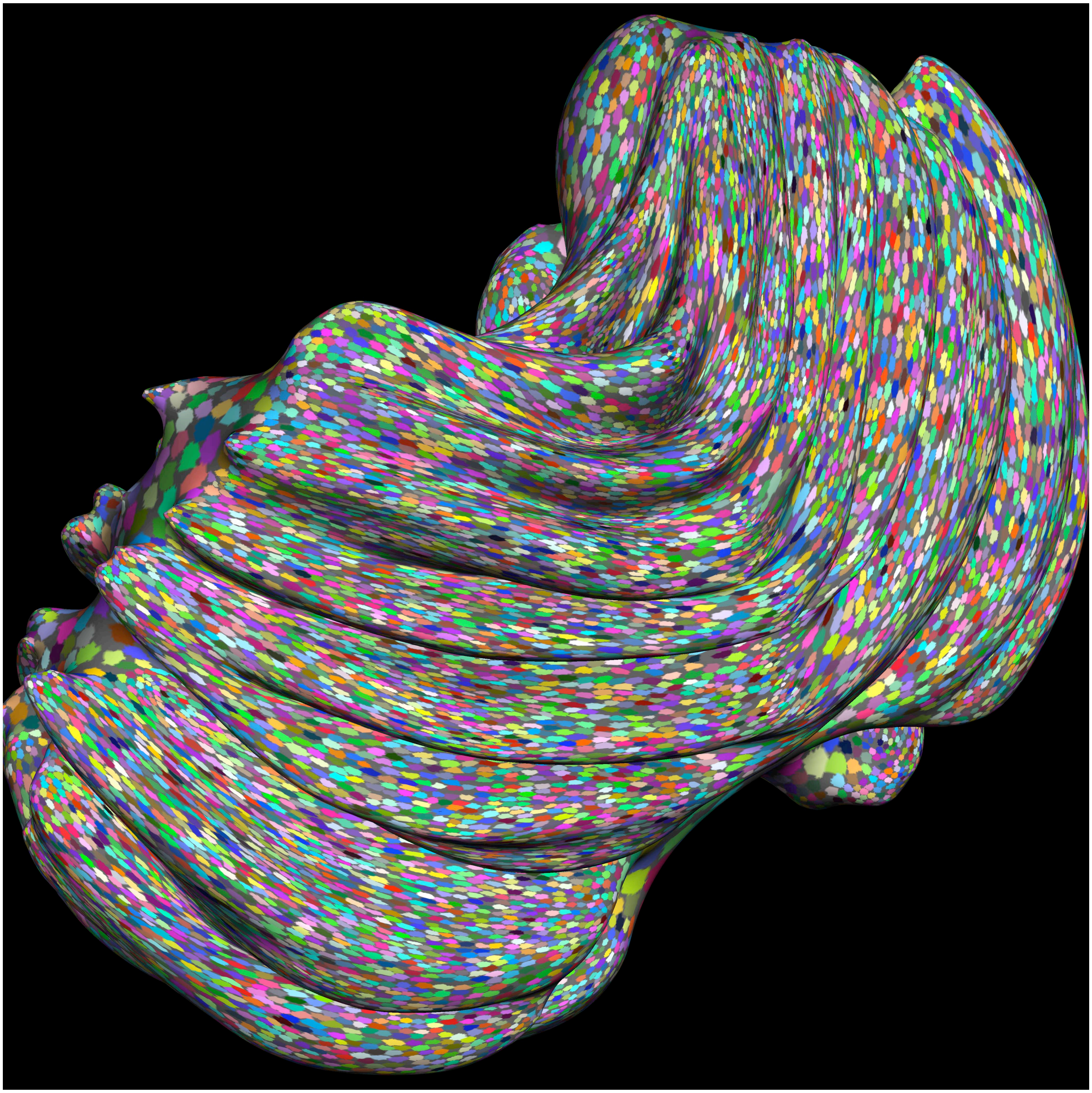

Figure S1. Approximate expected size of human cerebellar 'fractured somatotopy' patches. The simulated patches have been assigned random colors and painted onto the inflated surface of the human cerebellum in superior-posterior view. *In vivo* functional MRI scans of the human cerebellum at ultra-high fields should have sufficient resolution to be able to resolve these patches (e.g., using phase-encoded somatotopic mapping methods in the somatosensory cerebellum). Note that it is currently unknown whether a similar 'fractured map' organization extends beyond somatosensory regions in the cerebellum.

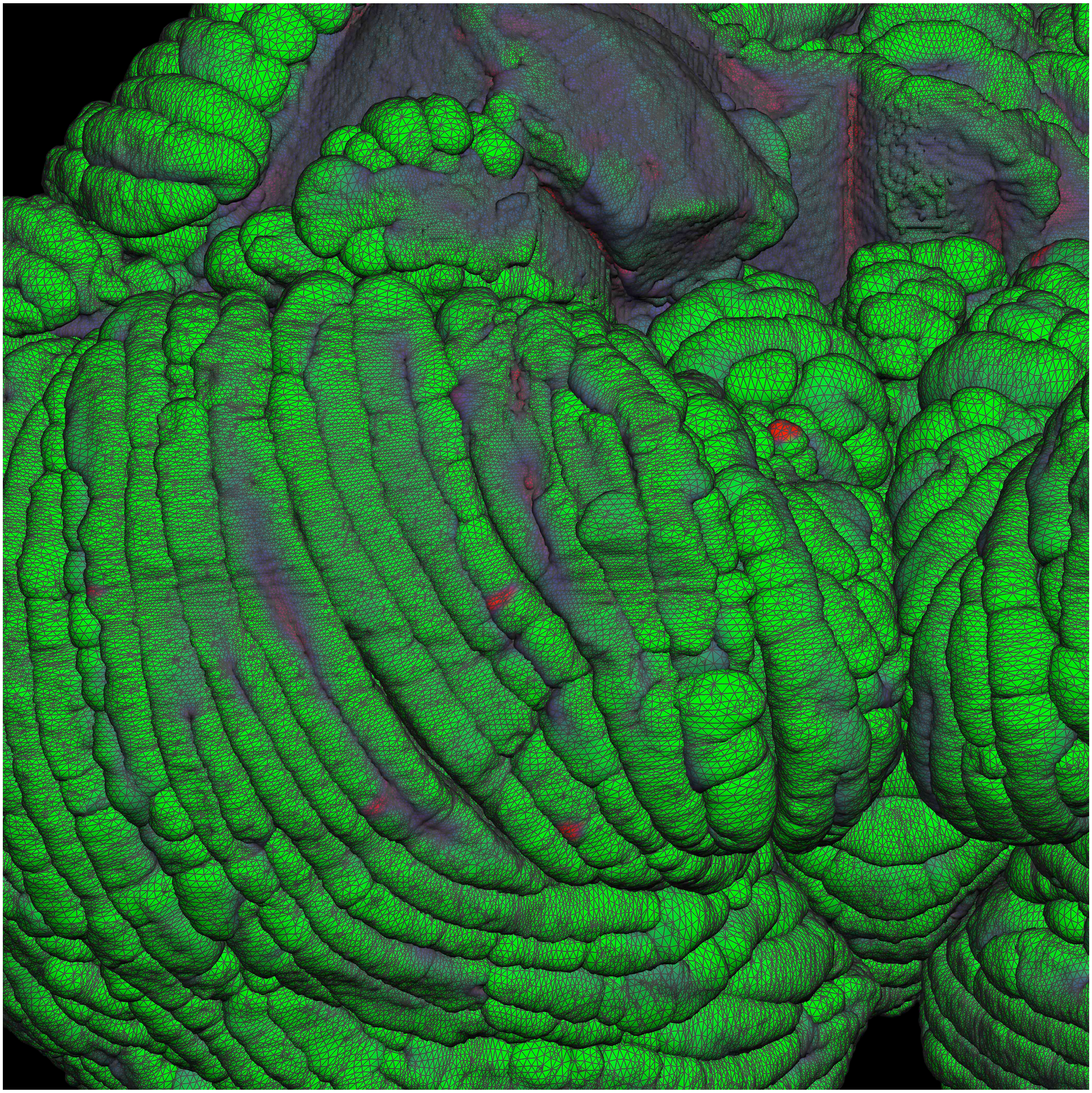

Figure S2. Inferior-anterior view of folded cerebellar surface reconstruction illustrating the fineness of the tessellated mesh. The paraflocculi (the 'tonsils') are visible at the lower middle right near the cerebellar midline. The right flocculus is visible in an inferior view near the top left, just below and slightly to the left of the cut surface of the middle cerebellar peduncle (blue, at middle top). At the far upper left, the lateral edges of the lobules in the anterior cerebellum are visible.

Supplementary Text

Materials and Methods

Specimen preparation and scan parameters. Cortical surface reconstruction methods developed for *in vivo* MRI images *(1)* were adapted to reconstruct the cortex of a formalin-fixed human cerebellum (62 year old female, cerebellum width: 96 mm, obtained post-mortem, de-identified prior to use in this study). The specimen was placed in a Fomblin-filled sealed acrylic cylinder, pressurized to approximately twice atmospheric pressure, and refrigerated and vibrated to induce air bubbles to dissolve. The sample was depressurized and warmed to room temperature several hours before scanning. The cylinder fit snugly within a 6 cm inside diameter quadrature RF coil, which was tuned with the sample in place using an RF analyzer. It was scanned on a 9.4T MR scanner (Agilent Technologies, Santa Clara, CA, USA) with short and long echo time (TE) standard 3D gradient echo (FLASH) sequences (9.4T; proton-density weighted (PD): flip=10deg, TE=3.7ms, TR=15ms; effective transverse relaxation time-weighted (T2*): flip=20deg, TE=18ms, TR=30ms; both scan sets used matrix: 512 x 340 x 340; isotropic 0.19 x 0.19 x 0.19 mm voxels; NEX=10; total time: 12h). Gray/white contrast was excellent in the T2* image and somewhat reduced in the PD image. Contrast between the granule cell and molecular layers of the cerebellar cortex in the T2* image varied somewhat across regions but was uniformly non-existent in the PD image. Since the bandwidth for both scan types was equivalent, B0-induced image distortion will be the same in both image sets. Our voxel width was roughly one-half the width of the long axis of the planar dendritic fields of human Purkinje cells.

Surface reconstruction. We updated the original FreeSurfer surface-reconstruction software utilities *(1)* to allow native use of 512^3 data sets (nominally 0.5 x 0.5 x 0.5 mm, but see below), improved anisotropic filtering, and adequate performance with much denser tessellations (free downloads of *csurf* for macOS 10.6.8 and higher, and linux CentOS 5.9 and higher are available at https://mri.sdsu.edu/sereno/csurf or http://www.cogsci.ucsd.edu/~sereno/.tmp/dist/csurf). The

cerebellum was reconstructed as a single closed surface without dividing it into left and right hemispheres.

Raw averaged T2* and PD images (Fig. 1, top row) were first masked to zero the nearly-zero Fomblin signal outside the sample. We then brightness-normalized the T2* image by dividing it by the PD image, further flattened the result with AFNI *3dUniformize*, and then brightness-inverted it so that white matter was lighter than gray matter, while keeping the region outside the cerebellum black (*brain.mgz*).

The final combined data set was then imported into *csurf* (but without downsampling it to 0.5 mm wide voxels, so no resolution was lost) and then anisotropically filtered: for a 7x7x7 voxel volume around each voxel, the plane of least brightness variation (perpendicular to one of 21 icosahedral search axes) was found, and then a 2D Gaussian was applied just in that plane (with a kernel full-width half maximum (FWHM) of 2.5 voxel widths = 0.40 mm) using a multi-threaded version of csurf *wmfilter*. Since this method only smooths in directions of low variance and since it is a 2D instead of a 3D operation, it blurs the high contrast boundaries of planar structures much less (Fig. 1, bottom left) than would a 3D Gaussian convolution with a similar FWHM.

Note that, in contrast to the situation with *in vivo* T1-weighted scans of the neocortex, where the gray/white matter border is characterized by substantial image contrast, the border between the *ex vivo* cerebellar white matter and the cerebellar molecular layer in our contrast-inverted T2*/PD-weighted scans has lower image contrast than the granule cell/molecular layer boundary (i.e., the location of the Purkinje cell layer). Another difference is that the gray/white matter border in the cerebellum approaches itself much more closely than does the neocortical gray/white matter border. Therefore, to obtain the most reliable initial 'white matter' segmentation (*wm.mgz*), we set the threshold for 'white matter' high enough so that it included the granule cell layer.

The segmented image was then filled (using csurf *fill*) by recursive region-growing (inside-out, outside-in, and inside-out) to generate a topologically nearly correct block of 'white

matter' voxels (*filled.mgz*). That 'white matter' image was then tessellated (using csurf *surf*, at native 190 micron wide resolution) to generate an initial surface estimate.

However, numerous small topological defects remained (e.g., "wormholes" connecting adjacent folia or opposite sides of the same folium). The 'white matter' image (*wm.mgz*) was therefore repeatedly hand-edited, re-filled, and re-tessellated to remove these defects to permit the surface to be unfolded. The resulting surface (4.6M vertices, 9.2M triangles, 25x data size of standard FreeSurfer neocortical hemisphere) was then unfolded (FreeSurfer 5.3 *mris_inflate*, with non-standard parameters [see *csurf* Expert Preferences] to accommodate the denser tessellation and larger amount of intrinsic curvature in the cerebellum) *(2)*. See SI Appendix, Figure S2 for a representation of the density of the surface mesh.

The initial folded surface estimate, roughly at the level of the Purkinje cell layer, was also transformed into a gray/white matter surface and a pial surface (Fig. 1, bottom right, yellow and green lines, and Fig. 3) using csurf *tksurfer* by setting the image criterion for zero image error either to a value just below the brightness of the white matter, or to a low value in order to cause the surface to settle near the pial surface, while including a vertexwise test that prevents surface self-intersection of the estimated surfaces of the tightly packed folia. The non-self-intersecting pial surface estimate was then further refined using the PD scan (csurf *tksurfer*). Finally, the surfaces were scaled to actual size (0.50/0.19 = 0.38 linear scale factor).

The surface was then cut into pieces (using csurf *tksurfer*) to allow it to be flattened without severe areal distortion using FreeSurfer 5.3 *mris_flatten (3).* The flattened representations were used for display purposes (but not areal measurements).

Measurement of area. Areal measurements are the sum of 'vertexwise area', which is calculated as the sum of 1/3 of the area of each surrounding triangular face of the cerebellar cortex. The surface was first corrected for shrinkage (see below). The surface regions covering the cut peduncles as well as small patches of bare white matter were excluded from the area sum. Those cuts were done on the inflated surface using the curvature coloring to indicate the folia ends, and then the cuts were transferred to the corresponding vertices on the pial surface (csurf *tksurfer*).

The pial surface was chosen for areal measurement because the pial surface has the highest contrast, making it the most definitive measurement. Also, it allowed direct comparisons to previous stereological estimates in the literature.

To make a direct comparison to the *in vivo* human data for the neocortex, we needed an estimate of the human neocortical pial surface area. This surface is routinely reconstructed in FreeSurfer with the *recon-all* pipeline. Using FreeSurfer 5.3 reconstructed human scans from our laboratory, the ratio in the neocortex between the gray/white surface area and the pial surface area was measured to be 1.21 (across 32 hemispheres). We therefore multiplied the average female standard "white" surface area taken from the literature by this factor before comparing it to our cerebellar pial surface area.

Correction for shrinkage due to fixation. From a careful longitudinal study of a single human head specimen *(4)*, the brain was found to rapidly expand by about 5% in volume upon removing it from the skull (and being post-fixed for one day in formalin). Then, over a period of 70 days (comparable to the fixation time of our specimen before scanning), the brain volume reached an asymptote of about 8% volume shrinkage compared to the first out-of-skull measurement. This is comparable to the 5-10% volume shrinkage estimates from many previous studies of initial out-of-skull volume to final post-fixed volume. Note that this means that the total shrinkage with respect to the *in vivo* condition is only about 3%. We did not have access to an *in vivo* scan of the brain of our specimen. However, if we assume an isotropic volume shrinkage of 3% for our specimen (from *in vivo* to post-fixed *ex vivo* conditions, equivalent to a ~1% shrinkage in linear dimensions), this will result in a ~2% underestimate of the surface area of the cerebellar pial surface. Before shrinkage correction, the area of the pial surface (minus surface regions covering bare white matter) was 1559 cm^2. After applying a linear scale factor of 1.01 to the 3D pial surface (3% increase in volume of cerebellar convex hull), we measured a final shrinkage-corrected cerebellar pial surface area of 1590 cm^2.

We applied the same correction for fixation-induced shrinkage to the cerebellar and neocortical areal estimates for the *ex vivo* macaque monkey surfaces.

Sample cerebellum size in relation to average human cerebellum. Two methods were used to estimate the size of our *ex vivo* specimen relative to an average human cerebellum.

First, we extracted the left and right cerebellar gray and white matter from the FreeSurfer fsaverage Destrieux atlas volume segmentation (aparc.a2009s+aseg.mgz). The protruding cerebellar peduncles were edited to be flush with the anterior- and superior-most extent of the cerebellar gray matter. The almost-flush peduncle cuts in our specimen were then touched up to match those fsaverage edits. The respective extracted volumes were as follows: fsaverage overall cerebellum volume male+female: 153 cm^3, our female specimen: 142 cm^3. After 3% volume shrinkage correction, the surface area of our specimen was 146 cm^3. This measurement does not rely on the details of image contrast, since the cerebellar convex hull has excellent contrast in both *in vivo* and *ex vivo* cases. This is comparable to other slightly differently bounded measurements in the literature (e.g., 134 cm^3 from *(6)*).

Second, we started with the estimate of the volume of the cerebellar gray matter from *(5)*, which was 118.9 cm^3 for male participants and 109.3 cm^3 for female participants. These volumes are comparable to previous reported volumes of 112 cm^3 *(7)* and 108 cm^3 (for a group of female participants *(8)*. Applying similar methods to a downsampled version of our data set yielded 118.5 cm^3. Note that these results have to be treated with some caution, as it is difficult to directly compare *in vivo* to *ex vivo* measurements of cerebellar cortical volume given differences in mechanisms of *in vivo* and *ex vivo* image contrast, and because the contrast differences have the potential to differentially affect voxel classification. Furthermore, the relatively low resolution used in *(6)* cannot resolve the extremely tight folding pattern of the folia -- therefore the small branches of the white matter embedded in gray matter were not removed.

Overall, however, these two methods suggest that the volume of our human cerebellum specimen is not at all untypical.

For comparison with the human data and with average monkey data, our shrinkage-corrected macaque monkey cerebellum specimen had a volume of 7.36 cm^3, which was comparable to estimates in the literature, e.g., 7.1 cm^3 in *(5)*.

Maximum voxel size capable of full recovery of cerebellar folia in humans. To determine this, we started with the final normalized, contrast-inverted T2*-divided-by-PD 3D data set and then downsampled it, using cubic interpolation, with AFNI *3dresample*. We then used the resulting images to re-reconstruct the surface at a variety of downsampled resolutions. There remained a number of tiny topological defects (small 'wormholes' bridging folia) in the reconstructed surfaces which we didn't manually correct since we didn't need to unfold the surface. These are unlikely to have substantially changed the surface area estimate since they often involve only one misclassified voxel, and since the resulting 'wormhole' is stretched into an extremely narrow strand with a much reduced surface area after the surface has been refined. A selection of the downsampling results were as follows:

0.19 mm -> 0.50 mm: 50% loss in surface area (2.6x voxel width), 0.4M vertices
0.19 mm -> 0.28 mm: 14% loss in surface area (1.5x voxel width), 1.9M vertices
0.19 mm -> 0.21 mm: 1% loss in surface area (1.1x voxel width), 3.7M vertices
0.19 mm (no downsample): 4.6M vertices

These results show that for the human cerebellum, a minor downsampling results in little areal loss, but that areal loss rapidly non-linearly accelerates, reaching 50% at only 2.6x our native voxel width.

One fine point is that any form of interpolation, by definition, results in some additional smoothing of an image because the values of multiple nearby voxels in 3D must affect each single voxel in the downsampled image, typically implemented as a 3D convolution kernel extending to nearest neighbor voxels or next-nearest neighbor voxels. This is particularly an issue when comparing gentle downsampling (e.g., 0.19 mm -> 0.21 mm, or 0.19 mm -> 0.28 mm). In order to make fair areal comparisons with the original surface, therefore, we very gently smoothed the original, non-downsampled 3D data with a 0.95 voxel-width FWHM Gaussian (with csurf *tkmedit*) and re-reconstructed the surface (and didn't correct 'wormholes' as with the previous downsampled reconstructions). This resulted in a surface with roughly the same number of vertices (4.6M) as the original surface, but 5.8% less surface area than our original surface

made from the non-smoothed data. We then used that surface area estimate to calculate percent loss in surface area for the downsampled reconstructions shown above.

Completeness of recovery of folia from macaque monkey scan. The cerebellar folia of the macaque monkey cerebellum are about one-third the width of the cerebellar folia in humans; but the voxel volume of the macaque monkey scan could only reduced by one-half (0.19 mm to 0.15 mm voxel width) in the present study. On the positive side, the macaque monkey cerebellum reconstruction was less computationally challenging than for the human cerebellum because the branching pattern of the folia in monkeys is much simpler. A main white matter strand emanating from the central white matter core typically branches just a few times in the macaque monkey cerebellum; in humans, by contrast, one main white matter strand may branch into 50 or 60 folia in crus I and II. Accurate initial recovery of the surface of all folia depends upon the FreeSurfer region-growing method (csurf *fill*) sequentially finding its way throughout every branch in the white matter tree without failures or 'leaks', which are much more likely in the human case, making the monkey cerebellum reconstruction somewhat less susceptible to non-optimal voxel size.

We inspected of every slice image intersected with the final surface reconstruction, to verify that we had in fact recovered every folium, hand-edited the few that were missed, and re-reconstructed the surface before measuring its area.

Nevertheless, given the suboptimal ratio between folia width and voxel size in the current macaque monkey data set, it is likely that the surface area of the macaque monkey cerebellum was slightly underestimated. A future study with even smaller voxels will be required to definitively address this question.

SI References

1. A.M. Dale and M.I. Sereno, Improved localization of cortical activity by combining EEG and MEG with MRI cortical surface reconstruction: a linear approach. *Jour. Cog. Neurosci.* **5**, 162-176 (1993).

2. A.M. Dale, B. Fischl, and M.I. Sereno, Cortical surface-based analysis I: Segmentation and surface reconstruction. *NeuroImage* **9**, 179-194 (1999).

3. B. Fischl, M.I. Sereno, and A.M. Dale, Cortical surface-based analysis II: Inflation, flattening, and a surface-based coordinate system. *NeuroImage* **9**, 195-207 (1999).

4. G. Schulz, H.J.A. Crooijmans, M. Germann, K. Scheffler, M Müller-Gerble, B. Müller, Three-dimensional strain fields in human brain resulting from formalin fixation. *J. Neurosci. Meth.* **202**, 17-27 (2011).

5. J.K. Rilling and T.R. Insel, Evolution of the cerebellum in primates: differences in relative volume among monkeys, apes and humans. Brain Behav. Evol. **52**, 308-314 (1998).

6. J. Diedrichsen, J.H. Balsters, J. Flavell, E. Cussans, and N. Ramnani, A probabilistic MR atlas of the human cerebellum. Neuroimage 46, 39-46 (2009).

7. N. Makris, J.E. Schler, S.M. Hodge, C. Haselgrove, M.D. Albaugh, L.J. Seidman, S.L. Rauch, G. Harris, J. Biederman, V.S. Caviness Jr, D.N. Kennedy, J.D. Schmahmann, MRI-based surface-assisted parcellation of human cerebellar cortex: an anatomically specified method with estimate of reliability. *Neuroimage* **25**, 1146-60 (2005).

8. N.J. Keuthen, N. Makris, J.E. Schlerf, B. Martis, C.R. Savage, K. McMullin, L.J. Seidman, J.D Schmahmann, D.N. Kennedy, S.M. Hodge, S.L. Rauch, Evidence for Reduced Cerebellar Volumes in Trichotillomania. *Biol. Psychiatry* **61**, 374-381 (2007).
